# Supplementary material for: The Impact of Focused Ultrasound in Two Tumor Models: Temporal Alterations in the Natural History on Tumor Microenvironment and Immune Cell Response
Source: Cancers (Basel). 2020 Feb 4;12(2):350. doi: 10.3390/cancers12020350 (PMC7072338; doi:10.3390/cancers12020350)
Supplement: Supplementary file 1 [file cancers-12-00350-s001.pdf]

Supplementary Materials: The Impact of Focused Ultrasound in Two Tumor Models: Temporal Alterations in the Natural History on Tumor Microenvironment and Immune Cell Response

Gadi Cohen, Parwathy Chandran, Rebecca M. Lorsung, Lauren E. Tomlinson, Maggie Sundby, Scott R. Burks and Joseph A. Frank

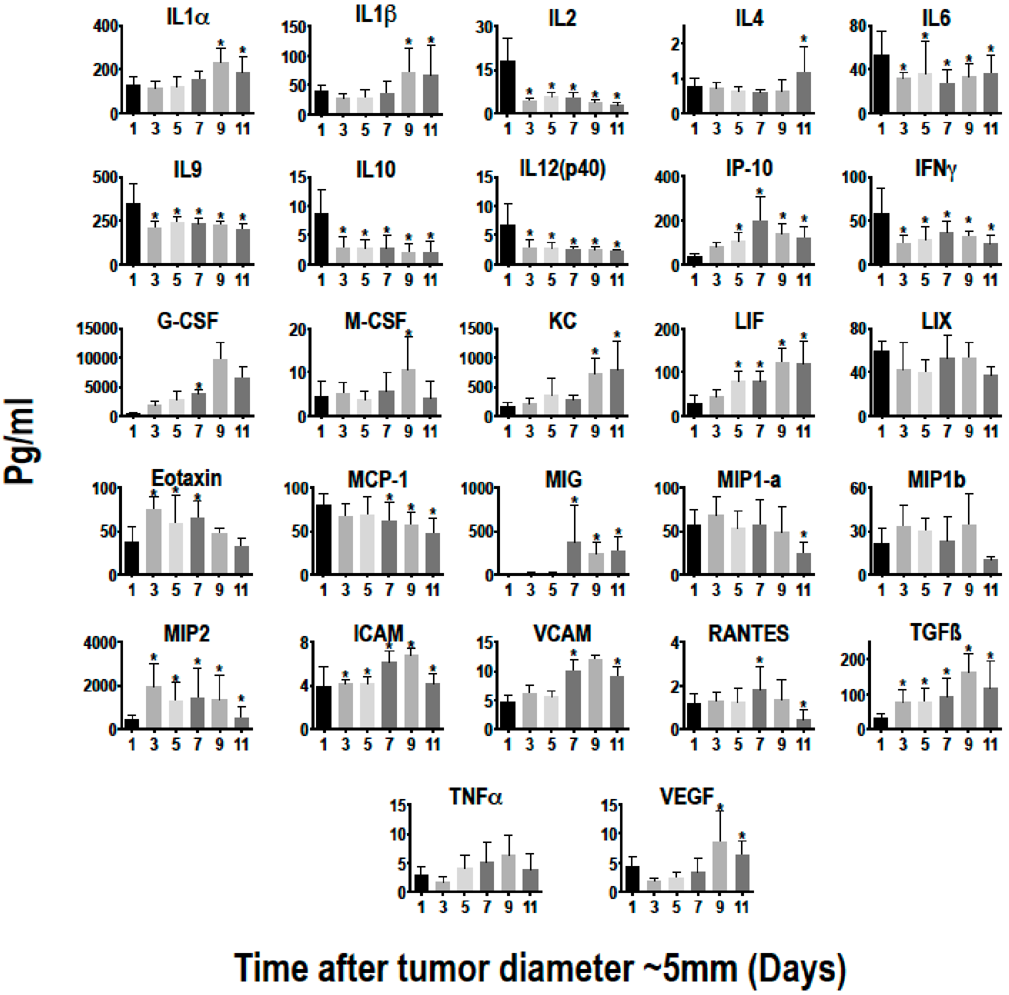

Figure 1. Quantitative values of CCTFs over time in mouse xenograft B16 melanoma tumor model. The y axis represents picograms (Pg) per milliliter; the x axis represents days after reaching ~5mm size in diameter. Asterisks indicate statistically significance compare to values detected on day 1 ($p < 0.05$; ANOVA).

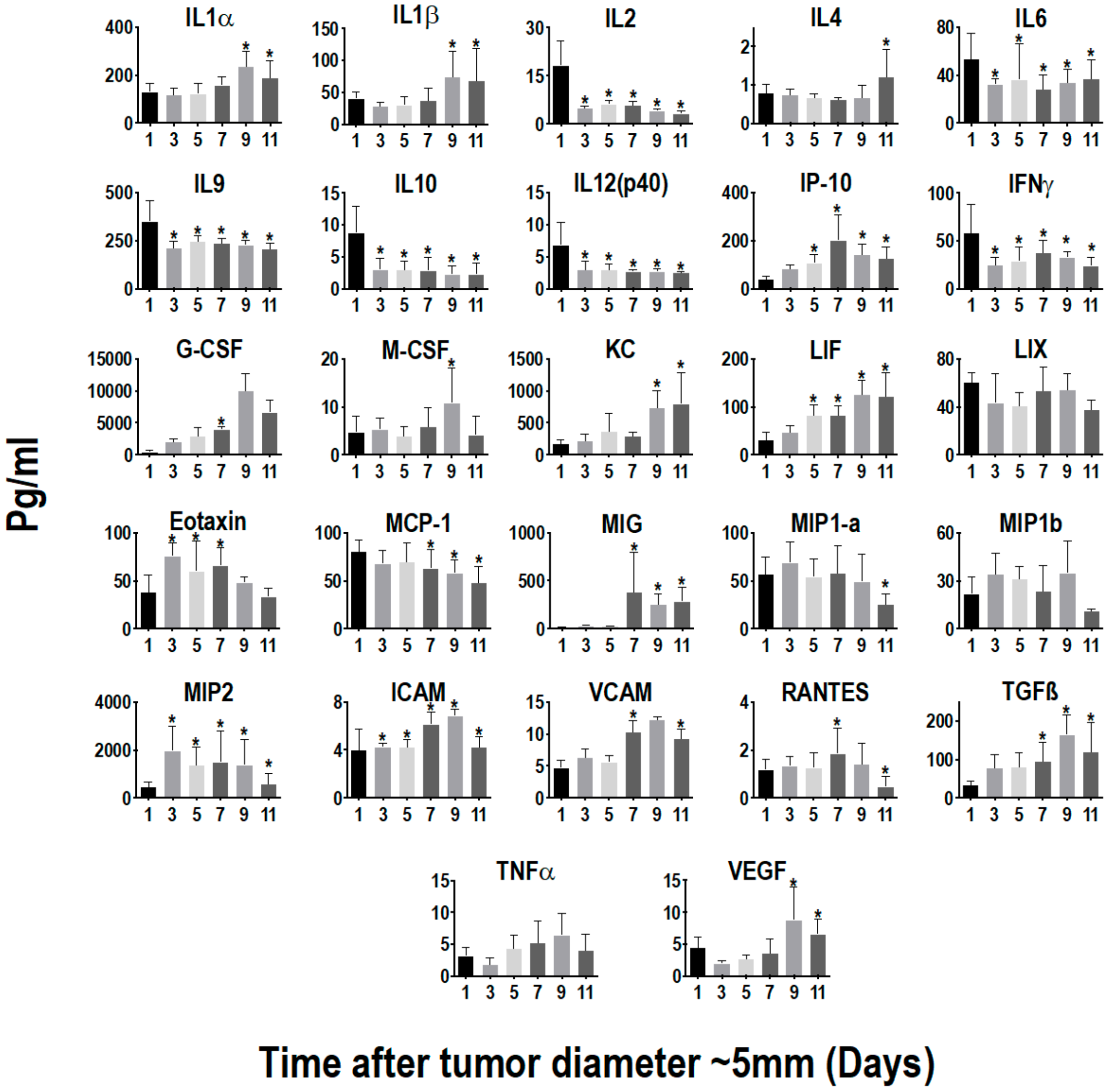

Figure 2. Quantitative values of CCTFs over time in mouse xenograft 4T1 breast cancer model. The y axis represents picograms (Pg) per milliliter; the x axis represents days after reaching ~5mm size in diameter. Asterisks indicate statistically significance compare to values detected on day 1 ($p < 0.05$; ANOVA).

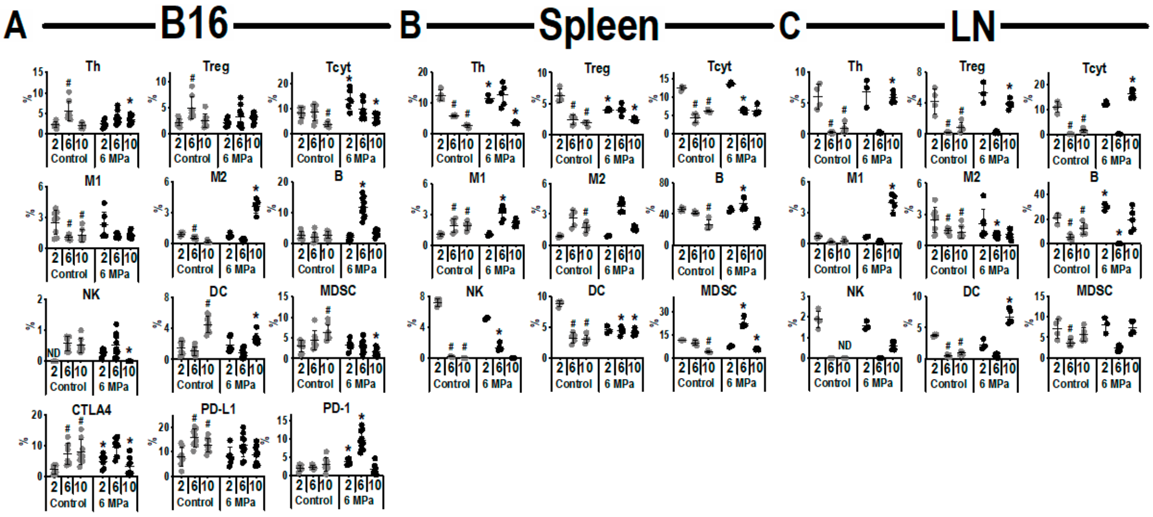

Figure 3. Immune cell profiles in B16 melanoma tumor bearing mice 24 hr following pFUS treatment on days 2, 6 or 10. Quantitative values of immune cell profiles over time of mouse B16 tumors (**A**) their spleens (**B**) and regional lymph nodes (LN; **C**), dissected 24 hr post-pFUS treatment. The y axis represents the relationship between each cell population to the total cells detected; the x axis represents 2, 6 or 10 days after reaching ~5mm size in diameter. Asterisks indicate statistically significance ($p < 0.05$; ANOVA) between total cells detected in pFUS-treated mice samples to time-matched controls. Pound indicate statistically significance ($p < 0.05$; ANOVA) between total cells number detected in samples of naïve untreated mice to naïve untreated controls on day 2.

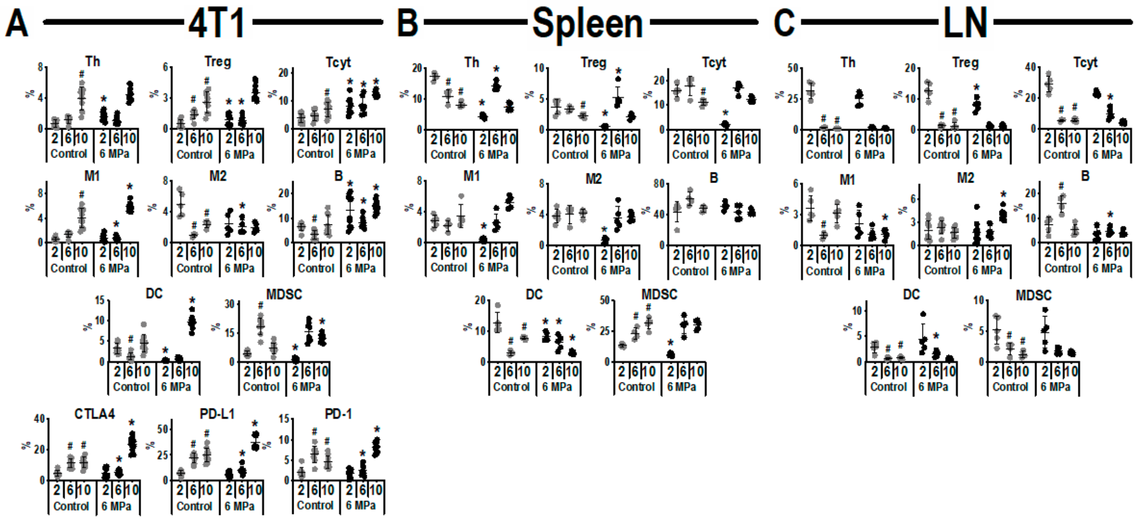

Figure 4. Immune cell profiles in 4T1 tumor bearing mice 24 hr following pFUS treatment on days 2, 6 or 10. Quantitative values of immune cell profiles over time of mouse 4T1 tumors (**A**) their spleens (**B**) and regional lymph nodes (LN; **C**), dissected 24 hr post-pFUS treatment. The y axis represents the relationship between each cell population to the total cells detected; the x axis represents 2, 6 or 10 days after reaching ~5mm size in diameter. Asterisks indicate statistically significance ($p < 0.05$; ANOVA) between total cells number detected in samples of pFUS-treated mice to time-matched controls. Pound indicate statistically significance ($p < 0.05$; ANOVA) between total cells number detected in samples of naïve untreated mice to naïve untreated controls on day 2.

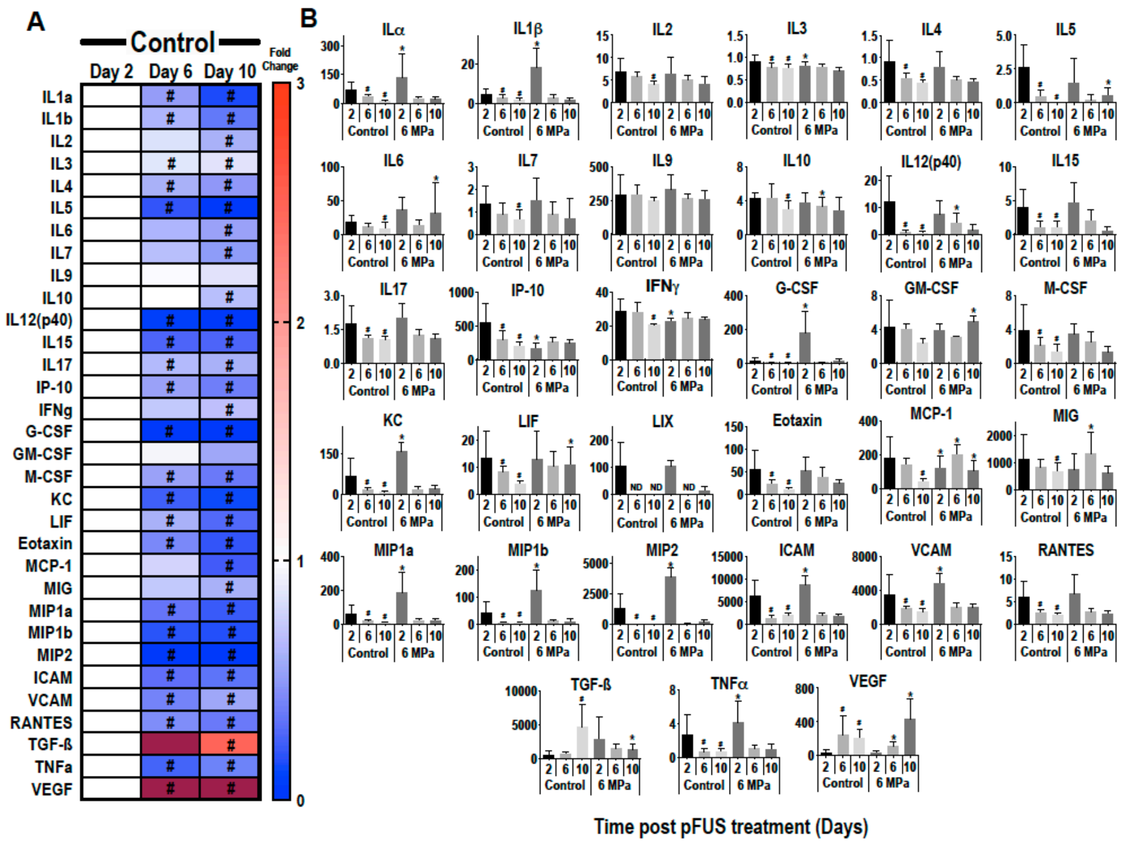

Figure 5. Proteomic changes of CCTFs and CAM over time in TME of untreated or pFUS-treated mouse xenograft B16 melanoma. **A**. Heat map demonstrate the normalized changes in CCTFs and CAM to Day 2 tumors un-sonicated B16 tumors compare to tumors on 6 and 10. Blue represent folds change less than 1. Red represent 1-3 folds change. Dark red represent folds change >3.1; **B.** Quantitative values of CCTFs and CAM over time of B16 melanoma tumors dissected 24 hr post sonicating on days 2, 6 or 10. The y axis represents picograms per milliliter; the x axis represents days after reaching ~5mm size in diameter. Asterisks indicate statistically significance ($p < 0.05$; ANOVA) between values detected in pFUS-treated tumors to time-matched controls. # indicate statistically significance ($p < 0.05$; ANOVA) between values detected in untreated tumors of naïve mice to naïve untreated controls on day 2.

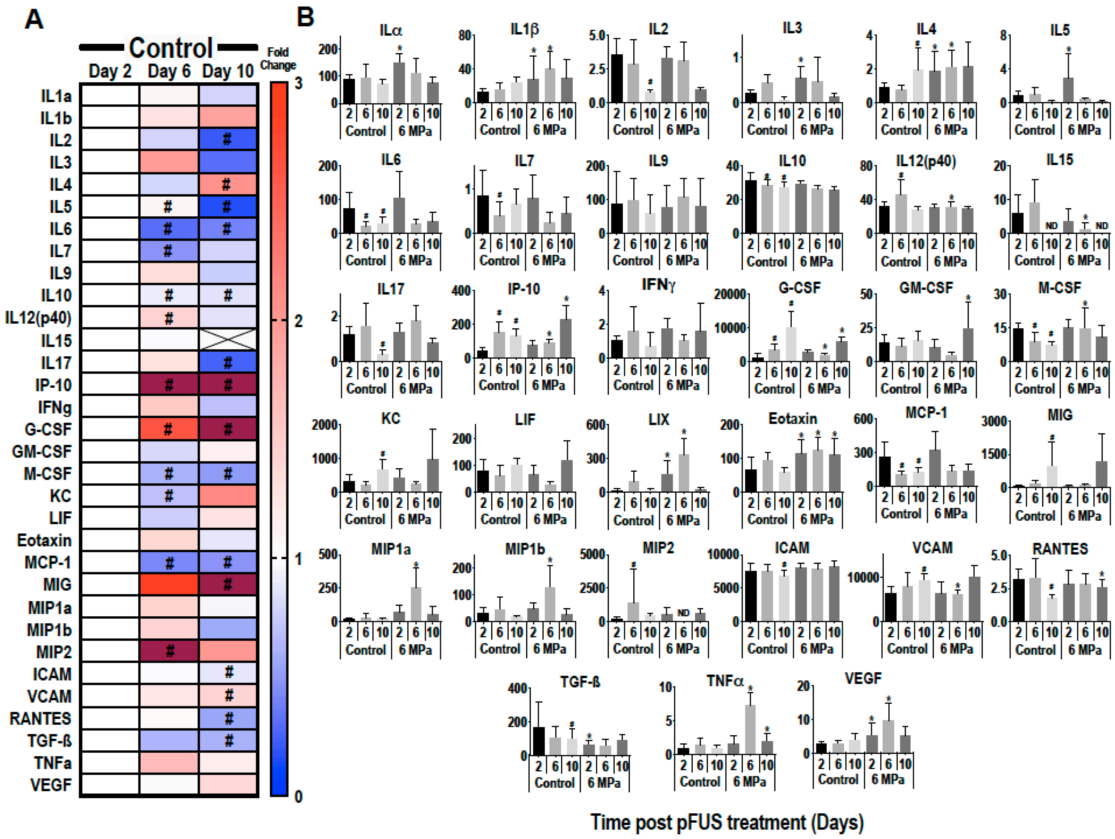

Figure 6. Proteomic changes of CCTFs and CAM over time in TME of untreated or pFUS-treated mouse xenograft 4T1 breast cancer. **A**. Heat map demonstrate the normalized changes in CCTFs and CAM to Day 2 tumors unsonicated 4T1 tumors compare to tumors on 6 and 10. Blue represent folds change less than 1. Red represent 1-3 folds change. Dark red represent folds change >3.1; **B.** Quantitative values of CCTFs over time of 4T1 breast cancer samples dissected 24 hr post sonicating on days 2, 6 or 10. The y axis represents picograms per milliliter; the x axis represents days after reaching ~5mm size in diameter. Asterisks indicate statistically significance ($p < 0.05$; ANOVA) between values detected in pFUS-treated tumors to time-matched controls. # indicate statistically significance ($p < 0.05$; ANOVA) between values detected in untreated tumors of naïve mice to naïve untreated controls on day 2.

Table 1. Detailed list of antibodies.

Item	Cat#	Clone	Working Conc. (µg/10^6 cells in 100 µl)	Isotype control	Cat#	Company	Clone
FITC anti-mouse CD3 Antibody	100204	17A2	1	FITC Rat IgG2b, κ Isotype Ctrl Antibody	400606	BioLegend	RTK4530
APC anti-mouse CD4 Antibody	100516	RM4-5	0.25	APC Rat IgG2a, κ Isotype Ctrl Antibody	400512	BioLegend	RTK2758
PE anti-mouse CD25 Antibody	102008	PC61	1	PE Rat IgG1, λ Isotype Ctrl Antibody	401906	BioLegend	G0114F7
PE anti-mouse CD8a Antibody	100708	53-6.7	0.25	PE Rat IgG2a, κ Isotype Ctrl Antibody	400508	BioLegend	RTK2758
FITC anti-mouse F4/80 Antibody	123108	BM8	0.25	FITC Rat IgG2a, κ Isotype Ctrl Antibody	400506	BioLegend	RTK2758
PE anti-mouse CD206 (MMR) Antibody	141706	C068C2	0.5	PE Rat IgG2a, κ Isotype Ctrl Antibody	400508	BioLegend	RTK2758
APC anti-mouse CD86 Antibody	105012	GL-1	0.25	APC Rat IgG2a, κ Isotype Ctrl Antibody	400512	BioLegend	RTK2758
Alexa Fluor® 488 anti-mouse/human CD45R/B220 Antibody	103225	RA3-6B2	2	Alexa Fluor® 488 Rat IgG2a, κ Isotype Ctrl Antibody	400525	BioLegend	RTK2758
Alexa Fluor® 647 anti-mouse CD335 (NKp46) Antibody	137628	29A1.4	0.5	Alexa Fluor® 647 Rat IgG2a, κ Isotype Ctrl Antibody	400526	BioLegend	RTK2758
FITC anti-mouse CD45 Antibody	103108	30-F11	0.25	FITC Rat IgG2b, κ Isotype Ctrl Antibody	400606	BioLegend	RTK4530
PE anti-mouse/human CD11b Antibody	101208	M1/70	0.25	PE Rat IgG2b, κ Isotype Ctrl Antibody	400608	BioLegend	RTK4530
APC anti-mouse Ly-6G/Ly-6C (Gr-1) Antibody	108412	RB6-8C5	0.25	APC anti-mouse Ly-6G/Ly-6C (Gr-1) Antibody	108412	BioLegend	RB6-8C5
PE anti-mouse CD11c Antibody	117308	N418	0.25	PE Armenian Hamster IgG Isotype Ctrl Antibody	400908	BioLegend	HTK888
APC anti-mouse CD152 (CTLA-4) Antibody	106310	UC10-4B9	1	PE Armenian Hamster IgG Isotype Ctrl Antibody	400912	BioLegend	HTK888
PE anti-mouse CD274 (B7-H1, PD-L1) Antibody	124308	10F.9G2	0.25	PE Rat IgG2b, κ Isotype Ctrl Antibody	400608	BioLegend	RTK4530
Alexa Fluor® 647 anti-mouse CD279 (PD-1) Antibody	135230	29F.1A12	0.25	Alexa Fluor® 647 Rat IgG2a, κ Isotype Ctrl Antibody	400526	BioLegend	RTK2758

© 2020 by the authors. Licensee MDPI, Basel, Switzerland. This article is an open access article distributed under the terms and conditions of the Creative Commons Attribution (CC BY) license (http://creativecommons.org/licenses/by/4.0/).
